# Dynamics and drivers of fungal communities in a multipartite ant-plant association

**DOI:** 10.1186/s12915-024-01897-y

**Published:** 2024-05-14

**Authors:** Veronica Barrajon-Santos, Maximilian Nepel, Bela Hausmann, Hermann Voglmayr, Dagmar Woebken, Veronika E. Mayer

**Affiliations:** 1 Department of Botany and Biodiversity Research, University of Vienna, Vienna, Austria; 2 Department of Microbiology and Ecosystem Science, Centre for Microbiology and Environmental Systems Science, University of Vienna, Vienna, Austria; 3 Doctoral School in Microbiology and Environmental Science, University of Vienna, Vienna, Austria; 4 Joint Microbiome Facility of the Medical University of Vienna and the University of Vienna, Vienna, Austria; 5 Department of Laboratory Medicine Division of Clinical Microbiology, Medical University of Vienna, Vienna, Austria; 6 Present Address: Plant Health and Environment Laboratory, Ministry for Primary Industries, Auckland, New Zealand

**Keywords:** Ant-plant mutualism, Fungal communities, *Azteca*, *Cecropia*, Insect-fungus interactions, Tropical ecosystems, Community dynamics, Ant-made patches

## Abstract

**Background:**

Fungi and ants belong to the most important organisms in terrestrial ecosystems on Earth. In nutrient-poor niches of tropical rainforests, they have developed steady ecological relationships as a successful survival strategy. In tropical ant-plant mutualisms worldwide, where resident ants provide the host plants with defense and nutrients in exchange for shelter and food, fungi are regularly found in the ant nesting space, inhabiting ant-made dark-colored piles (“patches”). Unlike the extensively investigated fungus-growing insects, where the fungi serve as the primary food source, the purpose of this ant-fungi association is less clear. To decipher the roles of fungi in these structures within ant nests, it is crucial to first understand the dynamics and drivers that influence fungal patch communities during ant colony development.

**Results:**

In this study, we investigated how the ant colony age and the ant-plant species affect the fungal community in the patches. As model we selected one of the most common mutualisms in the Tropics of America, the *Azteca-Cecropia* complex. By amplicon sequencing of the internal transcribed spacer 2 (ITS2) region, we analyzed the patch fungal communities of 93 *Azteca* spp. colonies inhabiting *Cecropia* spp. trees. Our study demonstrates that the fungal diversity in patches increases as the ant colony grows and that a change in the prevalent fungal taxa occurs between initial and established patches. In addition, the ant species significantly influences the composition of the fungal community in established ant colonies, rather than the host plant species.

**Conclusions:**

The fungal patch communities become more complex as the ant colony develops, due to an acquisition of fungi from the environment and a substrate diversification. Our results suggest a successional progression of the fungal communities in the patches during ant colony growth and place the ant colony as the main driver shaping such communities. The findings of this study demonstrate the unexpectedly complex nature of ant-plant mutualisms in tropical regions at a micro scale.

**Supplementary Information:**

The online version contains supplementary material available at 10.1186/s12915-024-01897-y.

## Background

Plants, ants, and fungi are key players in terrestrial ecosystems all over the world. While the role of plants is obvious, ants and fungi are often less understood. However, both groups have an enormous biomass [1, 2] and provide numerous important ecosystem functions. Ants turn and aerate the soil by digging nests and tunnels and contribute considerably to nutrient redistribution through scavenging large amounts of carrion and plant debris [3, 4]. Recent studies indicate that they are likely to be functionally non-replaceable in their foraging roles in tropical rainforests [4]. Fungi, with an estimated > 3 million species [5], are key players in soils being the dominant decomposers of the complex components of plant debris such as cellulose and lignin. While fungi are regularly found affecting the health of plants and animals as pathogens [6], they have also established mutualistic relationships with a wide range of organisms (e.g., lichens, mycorrhizae, insect-cultivated fungal gardens) [7–9].

In habitats where nutrient availability is notoriously low, like in tropical rainforests [10, 11], steady relationships between arthropods and fungi seem to be a recurrent survival strategy [12]. These interactions often have nutritional implications where arthropods either feed on fungi or indirectly benefit from their fungal enzymatic activity [9, 13–16]. In mutualistic associations, fungi are often rewarded with the dispersal of spores and constantly supplied with plant material as substrate [9, 17, 18]. Termites (Blattodea, Termitidae) and leaf-cutter ants (Hymenoptera, Formicidae) are examples for such mutualisms; the insects cultivate basidiomycetes for decomposing plant material they cannot digest themselves and feed on nutrient-rich fungal nodules [15, 19–22]. Similarly, ambrosia beetles (Coleoptera, Curculionidae) maintain complex fungal communities in their nests and use them as sole food source [16, 23].

In arboreal ants, and particularly in those that maintain mutualistic interactions with their hosting tree, a tripartite ant-plant-fungi association has been regularly documented [24–26]. Since the early twentieth century, slow-growing fungi, most of them from the order Chaetothyriales (Eurotiomycetes), have been repeatedly detected in the plant cavities used by the ants as nesting spaces (domatia) [27–30]. Unlike the mutualistic relationships between fungi and termites, leaf-cutter ants, or bark beetles, the purpose of the association between ants and domatia-inhabiting fungi is less obvious as the host plant already provides nutrient resources (e.g., food bodies or extrafloral nectar) to the ant colony [31–34]. By next generation sequencing, several investigations recently showed that, in addition to Chaetothyriales, there is a highly diverse fungal community inhabiting the domatia of different ant-plant associations [35–37]. These studies have shown that the fungal community composition varies spatially between differently used nest chambers of the same host plant and is also different from the surrounding soil.

**Fig. 1:**
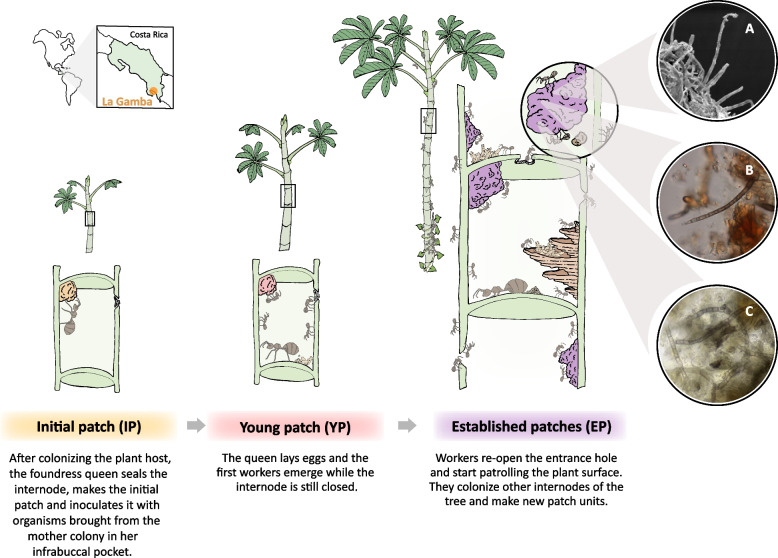
Graphic illustration of the *Azteca-Cecropia* association including ant-made patches from the three different ant colony development stages: initial patch (IP), young patch (YP), established patches (EP). Microscopic images of hyphae from established patches using scanning electron microscopy (**A**) and light microscopy (**B** and **C**). The map represents the geographic location of the sampling area of this investigation (La Gamba, Puntarenas, Costa Rica)

However, we are still lacking crucial information about the dynamics of fungal communities associated with ant-plant mutualisms. To study this, we chose the *Azteca-Cecropia* association as a model system. The interplay between the pioneer trees *Cecropia* spp. (Urticaceae) and their partner ants *Azteca* spp. (Formicidae, Dolichoderinae) is one of the most widespread and successful mutualisms in the Tropics of America [38]. *Azteca* ants defend their host plant against herbivores, phytopathogens and plant competitors [39–43]. In return, *Cecropia* trees provide ants with a nesting space inside the hollow stem (domatium) and plant-derived food bodies known as Müllerian bodies [44–46]. In this association, fungi, as well as bacteria and nematodes, are transgenerationally transmitted by the foundress queen who transfers these organisms to a self-made pile of parenchyma known as “patch” [25, 35, 47–50]. Several observations provide evidence of the importance of these patches for the *Azteca*-*Cecropia* association (Fig. 1). First, it was observed in 180 *Cecropia* saplings that the *Azteca* queens form the first patch before they start to lay their eggs [47]. Second, *Azteca* workers deposit plant tissue, ant feces, and ant corpses onto patches and constantly shape and manipulate them [24, 32, 47, 48]. Third, patch structures were found in almost every internode of the 93 colonies investigated, even in those with brood [48]. Last, none of the *Azteca* colonies inhabiting *Cecropia* stems from this study were found without patches in their nest.

Although the patches and the fungi they contain are recognized as permanent components in the *Azteca-Cecropia* mutualism [25, 31, 47, 48], nothing is currently known about the establishment of the fungal communities during the life cycle of ant colonies nor of the influence of the inhabiting ant species and the host-plant species. By analyzing amplicon sequence data of the ITS2 region, we investigated the fungal communities inhabiting patches of 93 colonies from three different *Azteca* species inhabiting *Cecropia* spp. Based on previous research [42, 47, 48, 51], we hypothesize that fungal diversity increases during ant colony development due to the increasing foraging and patrolling activity while the colony grows. This leads to the incorporation of spores or hyphal fragments from the environment into the patches. As ants are known to produce specific gland secretions that inhibit the germination of fungal spores and the growth of fungal hyphae [52–54], we expect a similar fungal community in patches from established colonies of the same ant species. And finally, we expect that the ant species plays a more significant role in influencing the composition of fungal patch communities than the plant species, given their evident dominance within the nesting environment [24, 35, 55]. Understanding the spatiotemporal dynamics of the fungal communities inhabiting the patches will help to unravel the purpose of these striking structures within the ant nests.

## Results

Amplicon sequencing of the ITS2 region from 93 *Azteca* ant colonies (Additional file 1: Table S1) yielded 1749 amplicon sequence variants (ASVs), of which 1280 ASVs (= 86.93% of total reads) were assigned to the kingdom Fungi, and more specifically, to 26 different fungal classes. Relative read abundance of each fungal taxon will be from now on referred to as relative abundance.

### Influence of the ant colony development on the fungal patch diversity

**Fig. 2:**
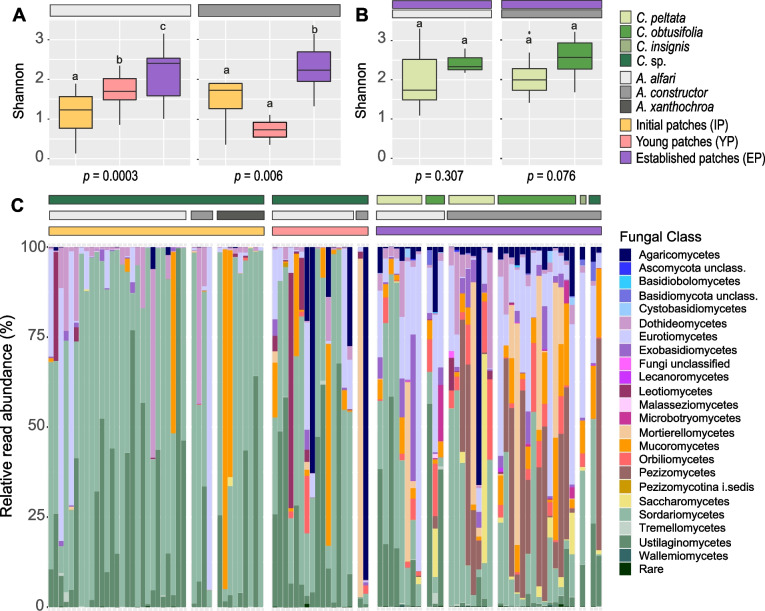
Diversity and taxonomic overview of fungal communities inhabiting ant-built patches. **A** Alpha diversity metrics (Shannon Index) of each ant species at different ant colony developmental stages (*A. alfari*: IP *n* = 27, YP *n* = 15, EP *n* = 12; *A. constructor*: IP *n* = 4, YP *n* = 2, EP *n* = 24). **B** Alpha diversity metrics (Shannon Index) of established colonies of each ant species inhabiting different plant species (*A. alfari*: *C. peltata n* = 8, *C. obtusifolia n* = 3; *A. constructor*: *C. peltata n* = 8, *C. obtusifolia n* = 14). In both cases (**A**, **B**), statistical comparisons (*p* < 0.05) by Kruskal–Wallis and Wilcoxon post hoc tests are shown. **C** Relative read abundances (%) of abundant fungal classes (> 0.5%) per ant colony, grouped by colony developmental stage, ant species and plant species. Low abundant taxa (< 0.5%) are merged as “Rare”

In the ant species *A. alfari* and *A. constructor*, we detected a significantly higher fungal alpha diversity in established patches than in the initial patches (*p* = 0.0008, and *p* = 0.0227, respectively) (Fig. 2A; Additional file 2: Tables S1-S2). Since *A. xanthochroa* colonies were only found at the initial stage, diversity comparisons could not be performed with this ant species. In initial patches of 40 *Azteca* spp. colonies, on average 4 ± 2 ASVs out of 31 ± 14 fungal ASVs accounted for at least 90% of total reads (Additional file 3: Table S1). Fungal communities of initial patches were dominated by ASVs assigned to classes Sordariomycetes (58.3% mean relative abundance), Ustilaginomycetes (20.8% mean relative abundance), Eurotiomycetes (8.9% mean relative abundance), and Dothideomycetes (4.9% mean relative abundance), except for three patches that were dominated by ASVs assigned to Mucoromycetes (Fig. 2C). These five classes represented 98.3% of total reads in all initial patches collected.

Young patches from 15 *A. alfari* colonies were significantly more diverse than initial patches and less diverse than established patches (Fig. 2A; Additional file 2: Table S1). In young colonies, classes Agaricomycetes and Leotiomycetes, which were not abundant in initial patches, increased to 8.3% and 5.7% mean relative abundance, respectively (Fig. 2C). Young patches from two *A. constructor* colonies showed a contrasting pattern: they harbored communities of slightly lower diversity than initial patches (Fig. 2A; Additional file 2: Table S2). This finding is most likely due to the notably low number of young *A. constructor* colonies included in the study.

The taxonomic composition of fungal patch communities from 36 established colonies revealed a high heterogeneity (Fig. 2C). Generally, established patches consisted of a few read-abundant ASVs and a high diversity of low abundant ASVs. On average, 15 ± 11 ASVs out of 189 ± 77 fungal ASVs accounted for at least 90% of total reads (Additional file 3: Table S1). In this ant colony developmental stage, we detected 11 different classes with more than 2.5% mean relative abundance, where Sordariomycetes, Eurotiomycetes, and Pezizomycetes showed the highest relative abundance (20.4%, 19% and 14.3%, respectively). In established patches, alpha diversity of fungal communities in each ant species did not vary between plant species (Fig. 2B; Additional file 2: Table S3-S4).

### Effect of the ant and the plant species on the fungal community composition

**Fig. 3:**
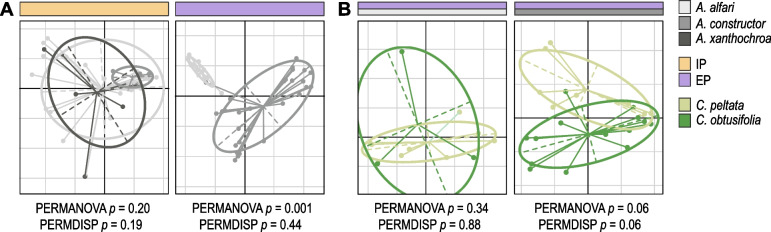
Beta diversity analysis of fungal community composition inhabiting ant-made patches represented by Principal Coordinate Analysis (PCoA) ordination using a Bray–Curtis dissimilarity distance matrix. **A** Comparison of different ant species per ant colony developmental stage (*A. alfari*: IP *n* = 27, EP *n* = 12; *A. constructor*: IP *n* = 4, EP *n* = 24; *A. xanthochroa*: IP *n* = 9). **B** Comparison of different plant species per ant species in established patches (*A. alfari*: *C. peltata n* = 8, *C. obtusifolia n* = 3; *A. constructor*: *C. peltata n* = 8, *C. obtusifolia n* = 14). Statistical analyses (*p* < 0.05) by PERMANOVA and PERMDISP tests are shown

To evaluate if the fungal community composition was significantly influenced by the ant or plant species, we performed beta diversity analyses based on Bray–Curtis distances among colonies (Fig. 3; Additional file 4). For initial patches, the PERMANOVA test showed no correlation between the fungal community variation and the ant species (*p* = 0.197). When comparing fungal community composition from established patches, we could detect a significant influence by the ant species (*p* = 0.001), but not by the plant species in neither *A. alfari* nor *A. constructor* colonies (*p* = 0.342 and *p* = 0.059, respectively). Since the sample size was notably unbalanced in most statistical analyses, additional PERMDISP and MiRKAT tests were performed in this study to provide sufficient statistical robustness (Additional file 4).

**Fig. 4:**
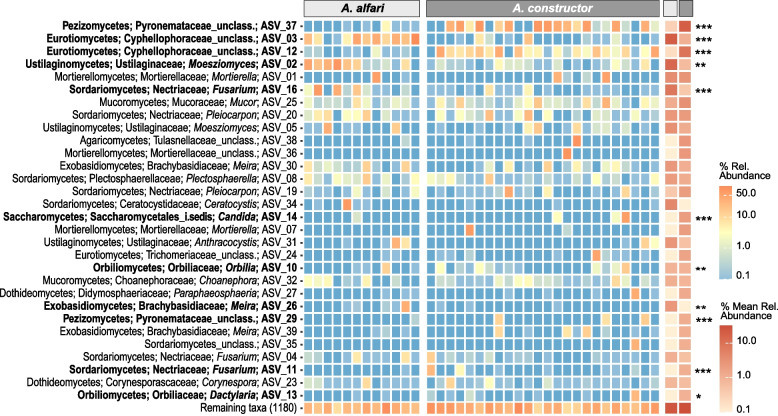
Heatmap depicting relative read abundances of the 30 most abundant fungal ASVs in patches from established ant colonies. Relative abundances of ASVs are shown per individual ant colony of each ant species (left, blue-orange) and as the average over all ant colonies per ant species (right, beige-terracotta). Relative abundances of ASVs between ant species are statistically compared by using DESeq2 analysis (adjusted *p* values: * < 0.05, ** < 0.01, and *** < 0.001). ASVs with significant different relative abundances between ant species are depicted in bold

As established patches showed the most diverse and distinct fungal communities, we used this developmental stage for further analysis at lower taxonomic levels. When looking at the 30 most abundant ASVs, we observed that certain ASVs were highly abundant and common particularly in colonies of the same ant species (Fig. 4). The most abundant ASV from *A. constructor* (ASV_37) was assigned to unclassified Pyronemataceae (Pezizomycetes, 19.8% mean relative abundance), yet, this ASV, and the family it belonged to, was present at only very low frequencies in patches from *A. alfari* (0.3% mean relative abundance) (adjusted *p* < 0.0001). Contrarily, ASV_02 belonging to the genus *Moesziomyces* (Ustilaginomycetes, Ustilaginaceae) was significantly more abundant in *A. alfari* (14.6% mean relative abundance) than in *A. constructor* (0.7% mean relative abundance) (adjusted *p* = 0.0012). Moreover, the second and third most abundant ASVs (ASV_03 and ASV_12), which belonged to two separate clusters of the Cyphellophoraceae family (Eurotiomycetes, Additional file 5: Fig. S1) [25, 47, 56–59], were significantly more predominant in one of the two ant species (adjusted *p* = 0.0003, and, adjusted *p* < 0.0001, respectively).

### Frequent fungal taxa in the patches and their dynamics over ant colony age

**Fig. 5:**
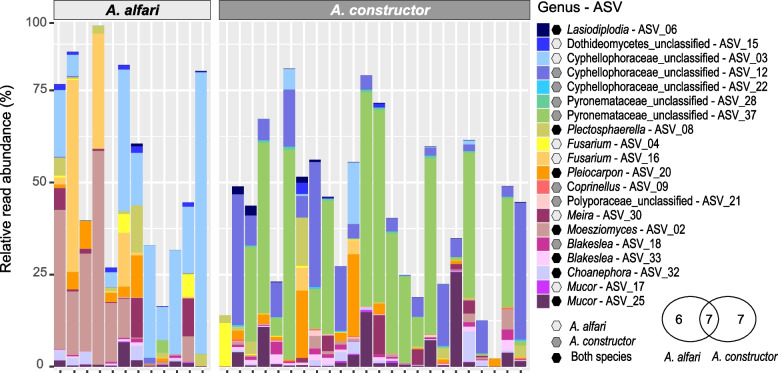
Taxonomic distribution of frequent fungal ASVs (present in more than 50% of colonies per ant species with a mean relative read abundance of > 0.05%) in proportion to the overall fungal diversity (100%) detected in patches from each established colony. In the legend, ASVs that were defined as frequent only in *A. alfari* are indicated with light grey hexagons, ASVs defined as frequent only in *A. constructor* with dark grey hexagons, and ASVs defined as frequent in both ant species with black hexagons. Venn diagram shows the number of ASVs that are frequent in either one or both ant species

To determine which fungal taxa are widely distributed in *Azteca-Cecropia* patches and how they change with ant colony age, we first searched for frequent fungal ASVs among all established colonies in each ant species. Frequent ASVs were defined as those that were present in at least half of the samples of each ant species with a mean relative abundance of at least 0.05%. Only 13 and 14 ASVs were detected as frequent in *A. alfari* and *A. constructor* colonies, respectively, from which 7 ASVs were frequent in both ant species (Fig. 5). Frequent ASVs accounted for a mean relative abundance of 54.96% in patches of *A. alfari* colonies and 39.76% in patches of *A. constructor* colonies. Among others, ASVs belonging to the genus *Fusarium* (ASV_04 and ASV_16, Sordariomycetes, Nectriaceae) were present in both ant species, but they were only defined as frequent in *A. alfari* patches.

**Fig. 6:**
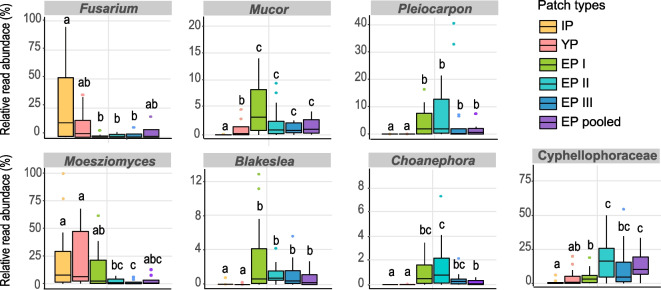
Relative read abundance (%) of selected genera encompassing frequent ASVs from patches of *Azteca* spp. Comparisons are made among ant colony development stages and tree sections within established colonies (initial patches, IP *n* = 40; young patches, YP *n* = 17; upper internode patches, EP I *n* = 9; intermediate internode patches, EP II *n* = 10; lower internode patches, EP III *n* = 6; all internode patches, EP pooled *n* = 11). Statistical comparisons are calculated by Kruskal–Wallis and Wilcoxon post hoc test (*p* < 0.05)

After defining the frequent fungal ASVs, we investigated whether the relative abundance of the genera they belong to varied among patch types (colony developmental stages and tree sections) in all *Azteca* sp.-*Cecropia* sp. colonies jointly (Fig. 6; Additional file 6). While ASVs belonging to *Fusarium* (Sordariomycetes, Nectriaceae) were predominant in initial patches, their relative abundance significantly decreased in patches of established colonies (EP I, EP II and EP III). *Moesziomyces* ASVs (Ustilaginomycetes, Ustilaginaceae) were notably abundant in initial patches, young patches and patches from the upper part of the tree in established colonies (EP I). *Mucor* (Mucoromycetes, Mucoraceae) and *Blakeslea* ASVs (Mucoromycetes, Choanephoraceae) presented an especially high relative abundance in upper internodes patches compared to patches from other tree sections and earlier colony developmental stages. Other ASVs belonging to Cyphellophoraceae family (Eurotiomycetes) considerably increased in relative abundance in patches from several established colonies, especially in the middle and most active part of the tree (EP II, 16.2% mean relative abundance) where brood and queen are typically found. Similarly, *Pleiocarpon* (Sordariomycetes, Nectriaceae) and *Choanephora* (Mucoromycetes, Choanephoraceae) ASVs were significantly more abundant in established than in initial patches.

## Discussion

### The fungal diversity in the patches increases with the ant colony development

**Fig. 7:**
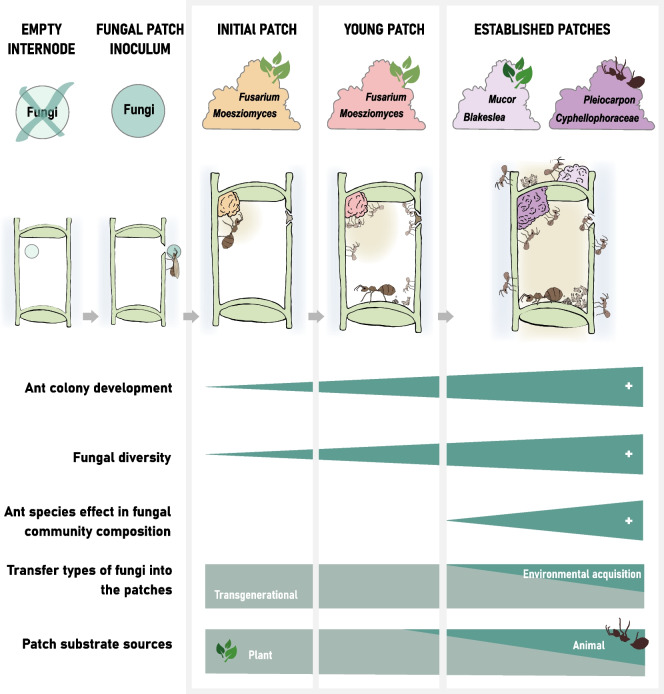
Conceptual illustration showing the successional progression of the fungal communities inhabiting ant-made patches from the *Azteca-Cecropia* association driven by the ant species, the diversification of substrates and the transfer types of fungi

We showed that the fungal communities become more complex as the ant colony grows, indicated by a significant increase in alpha diversity from initial to established patches. This may be due to two factors (Fig. 7): changes in the patch substrate during colony development and an increasing transfer of fungal spores from the environment. First, after entering the domatia, the founding queen makes the initial patch by scratching parenchyma tissue from the inner domatia wall and inoculates it with patch particles she brought from the mother colony [47]. The cellulose-dominated substrate appears to cause a bottleneck in the early establishment of the fungal patch community. This phenomenon has already been observed in the bacterial community of the same patches [48] and was explained by the N-deficiency of the parenchyma which favors the growth of organisms that are adapted to the low nitrogen content [60]. As the colony develops, ant workers make new patch structures in almost every internode they colonize. Additionally, ant workers diversify the substrate by adding different plant material such as trichomes and by depositing their feces and the carcasses of dead nestmates and insect prey onto those patches [25, 32]. The subsequent creation of more diverse micro-niches in the patches of established colonies enhance the development of a more complex community. Second, the vertical transmission of microorganisms by the founding queen is followed by an environmental acquisition through: (i) ant-workers patrolling and foraging on the host-plant surface [43, 51], (ii) opportunistic patch visitors such as dipteran larvae and mites [61, 62], and (iii) the air flow via the domatium entrance. While some fungi may indeed find a suitable niche in the patch environment, others may be inhibited by the high volatile concentration [63] or the fungicidal gland secretions [52–54] and remain as spores in the so-called microbial seed bank [64]. It is important to note that the widely used DNA-based identification approaches, such as the one used in this study, include both the active and the dormant fungal communities inhabiting the patches [65].

### Frequent fungal genera differ between initial and established patches

The change in relative abundances of the most frequent fungal genera across the different stages of the ant colony development indicates a successional progression within the fungal patch community over time (Fig. 7). ASVs belonging to the ubiquitous and fast-growing genera *Fusarium* (Sordariomycetes, Nectriaceae) [66] and *Moesziomyces* (Ustilaginomycetes, Ustilaginaceae) [67] were dominant in initial and young patches of all *Azteca* sp.-*Cecropia* sp. colonies investigated. Given their typical saprotrophic feeding strategy [67–69], members of these groups may be able to initiate organic matter decomposition processes in cellulose-dominated patches of early stages of the ant colony. Apart from the *Azteca-Cecropia* complex, *Fusarium* was detected in domatia of the myrmecophytic plants *Acacia drepanolobium* from Africa [37] and *Myrmecodia beccari* from Australia [36]. However, the authors did not distinguish between initial and established patches.

In the upper and younger internodes of established colonies, cellulose is also the dominant substrate, but in contrast to the initial patch where the foundress queen is the only ant in the internode, there are many more ants around. The secretions and behavior of the ant workers may cause a shift in fungal taxa as a significantly lower relative abundance of *Fusarium* sp. ASVs and a higher relative abundance of *Mucor* sp. ASVs (Mucoromycetes, Mucoraceae) and *Blakeslea* sp. ASVs (Mucoromycetes, Choanephoraceae) were detected.

In the middle and the basal internodes of established colonies, carcasses of dead nestmates are additionally added to the patches and probably used as substrate. The most prevalent ASVs in this tree section belong to Cyphellophoraceae (Eurotiomycetes, Chaetothyriales) and the genera *Pleiocarpon* (Sordariomycetes, Nectriaceae) and *Choanephora* (Mucoromycetes, Choanephoraceae). While *Pleiocarpon* and *Choanephora* have never been found in any ant-plant-association investigated so far, Cyphellophoraceae are known from many other ant-plant associations all over the tropics worldwide [24, 25, 27, 31, 36, 37]. The finding that Cyphellophoraceae are most abundant in established colonies, particularly in the stem regions of the nurseries [42], suggests a steady and direct ecological relationship between this particular group of fungi and the ant colony.

### Chaetothyriales fungi and their potential ecological roles in the patches

Microscopic examination of many ant-plant associations and subsequent cultivation identified Chaetothyriales as the most conspicuous and abundant fungal inhabitants of the domatia [25, 27, 30, 70]. The Chaetothyriales ITS sequences from the data set in the present study cluster in a monophyletic clade of uniquely domatia-inhabiting Chaetothyriales from Africa, Asia, and the Americas. Their frequent and exclusive occurrence in geographically distant ant-colonized domatia of ant-plant mutualisms studied so far worldwide [27, 30, 31, 70], as well as their reduced genome size compared to free-living Chaetothyriales strains [33], indicates an evolutionary advantage of vertical transmission and strongly suggests a mutualistic association with the ants [31].

Since the genomes of ant-associated Cyphellophoraceae lack genes for cellulose-active enzymes and other important polysaccharide lyase families [33], they are not major polysaccharide degraders as previously thought [32]. Their low abundance in the early stages of the colony could be explained by the fact that they need to rely on cross-feeding interactions with the fungal and bacterial network in the patches when cellulose is the main substrate. Such microbial network is still not developed in freshly made patches.

Until now, the roles of ant-associated Cyphellophoraceae have been related with secondary nutrition for the ant larvae [71], nutrient recycling [32], putative antimicrobial effects [33], and bio-filtration of the domatia air to remove toxic substances [63] that are produced by ants for communication [72] and diseases control [73]. Despite the efforts of many authors, an in-depth understanding of the ecological functions of Chaetothyriales as well as of the entire fungal community in the nests of ant-plant mutualisms remains elusive. Isolating and physiologically characterizing them will be a crucial step in the understanding of their ecology and activity in this specific environment.

### The ant species plays an important role in shaping the fungal patch communities

Despite the observed high inter-colony heterogeneity, the fungal community composition in patches of established colonies is significantly influenced by the *Azteca* species (Fig. 7). Although most fungal ASVs were found in both ant species, several prevalent ASVs showed higher relative abundance in patches of either *A. alfari* or *A. constructor*. In fact, both ant species differ in their behavior and the patches they build, thus creating different habitats [51]. *A. constructor* workers are more aggressive towards intruders than *A. alfari* and patrol the plant surfaces of *Cecropia* more often [51], which could increase the transfer of spores into the patches. *A. alfari* forms flat, dry and crumbly patches, whereas *A. constructor* forms larger, three-dimensional, and moist patches that reach anoxic conditions [60]. Although both ant species co-occur in the same geographical area, they seem to successfully develop in distinct environments and plant species. While established *A. constructor* colonies are regularly observed inhabiting *C. obtusifolia* in shady, humid, and steep locations close to streams and surrounded by dense vegetation, established *A. alfari* colonies are more often associated with *C. peltata* in hot, dry, and open areas such as river banks or road sites [51]. Despite the trend of finding more regularly each *Azteca* species in a particular *Cecropia* species, the fungal community composition in each ant species was not significantly affected by the plant species. These findings combined with the observed ability of the ant colony to modulate its nesting space [32, 35, 43, 47], suggest a pivotal role of the ants in influencing the microbial community in the patches.

### Open questions and hypothesis of the potential ant-plant-fungi interactions

After disentangling the dynamics and drivers of fungal communities inhabiting *Azteca-Cecropia* patches, the next questions are as follows: To what extent are the ants actively shaping the fungal communities in the patches? Do these communities provide a benefit to the ant colony, and if so, how? So far, we detected differential read abundances of frequent fungal groups among ant colony developmental stages and tree sections. However, whether such differences are related to the capability of the ant colony to promote or inhibit the growth of fungi remains unknown. Leaf-cutter ants and fungus-growing termites cultivate specific fungal symbionts in their nests while detecting and eliminating adverse fungal species [15, 74–77]. This does not seem to be the case with *Azteca* ants. Our finding of high heterogeneity in established colonies suggests that *Azteca* ants are either flexible or incapable of controlling which organisms are present in their patches. Several scenarios could explain why efficient screening has not evolved: (1) the *Azteca* ants are not affected by the presence of commensals in the patches as long as the beneficial fungi like Chaetothyriales can develop, (2) the ant colony is not adapted to a single fungus but to a fungal network, or (3) the patches provide a highly complex repertoire of niches that overcome the screening capabilities of the ants.

Compared to leaf-cutter ants and termites, ambrosia beetles are known to promote the growth of their diverse fungal partners by the colonization of ethanol-rich decaying trees [16, 78]. Similarly, *Azteca* ants could select for certain functionalities or metabolisms by modulating the addition of substrate to the patches, by producing volatiles that they usually use for pathogen defense, or by enlarging the entrance holes and thus, altering the ventilation in the domatia. However, such behaviors could still allow the growth of commensal or even harmful fungi that manage to adapt to these environmental conditions.

*Azteca* ants receive nutrient-rich food (Müllerian bodies) provided by *Cecropia* and honeydew produced by scale insects [34, 44, 46, 79]. Therefore, we would expect fungi to be used as a additional food source for ant larvae only when food bodies are scarce or if specific nutrients are not available in the regular food sources, as it has been shown in previous studies of other ant-plant mutualisms [47, 71]. Determining whether the *Azteca-Cecropia* association is indeed a “primitive” farming system, as recently suggested by Biedermann and Vega (2020) for ant-plant associations in general [9], requires a more comprehensive understanding of the ecological interactions among the organisms co-occurring in the *Azteca*-*Cecropia* ecosystem.

## Conclusions

The fungal communities in the *Azteca-Cecropia* association are characterized by a large diversity and high heterogeneity among colonies. A reason for this diversity is the combination of different vectors and modes of transmission affecting the fungal community: (i) vertical transmission of fungi from the queen’s mother colony, (ii) environmental acquisition of fungi from the plant surface through patrolling and foraging by the ant workers, and (iii) environmental acquisition of fungi through other arthropods such as flies and mites living in the patches of established ant colonies. Despite the high heterogeneity between colonies, the ant species significantly influences and shapes the fungal community in the patches. The ant colony seems to act as a keystone for the organisms co-habiting within the nest [48, 60], whereas the plant-host only provides the patch environment. Certainly, not all fungi in this association are symbionts, and even fewer are mutualists. A key aspect of future studies must be the development of a method to distinguish which groups are present as spores and which are present as mycelium. This would provide important information about which fungi are directly associated with the ant colony.

However, it is still a difficult task to elucidate their ecological relationships. What Six and Klepzig [80] pointed out for the bark beetle-fungus mutualism, that it is “notoriously difficult to manipulate in controlled experiments”, also applies to the *Azteca*-*Cecropia*-fungi association and leads to a lack of understanding of their interactions. Not only greenhouse experiments but also field experiments have failed, as ants abandon the manipulated domatia [24]. At the moment, instead of controlled experiments, we can only rely on careful observation and molecular analysis to elucidate the role of the fungal community in the patches of ant-plant associations.

## Methods

### Study site and sample collection

Samples were collected in the conservation zone ACOSA (*Área de Conservación Osa)* near the Tropical Field Station La Gamba in Puntarenas, Costa Rica (08° 42′ 03″ N, 083° 12′ 06″ W, 70 m a.s.l.). For this investigation, 93 *Azteca* ant colonies (*A. alfari*, *A. constructor* or *A. xanthochroa*) inhabiting 68 *Cecropia* trees from three species (*C. peltata*, *C. obtusifolia*, or *C. insignis*) were sampled next to roads, creeks, lowland forests, and pastures. Identification of ant species was performed based on the morphology of the ant colony and queen following the *Azteca* species descriptions [38, 81]. *Cecropia* species were identified by leaf characteristics [82].

After transversally opening *Cecropia* stems, ant-built patch samples were collected from the colonized internodes (domatia) by removing the whole patch material found in the stem with a dental probe. Immediately after, the patch material was transferred into RNA-later solution until further processing. Patch samples were classified in three categories based on the developmental stage of the ant colonies (Fig. 1). Initial (IP) and young (YP) patches were regularly analyzed individually, as these colonies only contained a single patch. Patches stemming from domatia of the same established ant colony (EP) were generally pooled. The patches from two colonies of the same ant species were pooled in eight samples due to an insufficient amount of patch material (*A. alfari* IP, *n* = 1; *A. alfari* YP, *n* = 3; *A. alfari* EP, *n* = 1; *A. constructor* YP, *n* = 1; *A. constructor* EP, *n* = 1; *A. xanthochroa* IP, *n* = 1) [83]. To investigate the fungal community variation within an established ant colony, tree stems from 17 established colonies were divided in three transverse sections based on the characteristics of the domatia and then, its patch material was collected separately (Additional file 6).

In the area of sampling, the abundance of the different *Azteca* and *Cecropia* species was notably uneven. For instance, *A. xanthochroa* colonies were only detected in an initial developmental stage and *Cecropia insignis* plants were rarely found. Since the ant species was only confirmed after collecting the plant, we were unable to obtain the same number of samples per each individual group. Additionally, we were only able to identify the plant species in established ant colonies since the distinctive leaf characteristics were not visible in younger plants. In Additional file 1, an overview of the number of colonies collected per ant species, plant species, and ant colony developmental stages is provided.

## Molecular analysis

In total, 120 patch samples stored in RNA-later solution were washed twice with a phosphate buffer (pH 8.0) by centrifuging the patch material for 1 min at 14,000 rpm. DNA was extracted from patch samples with an adapted phenol–chloroform based extraction protocol with three rounds of mechanical lysis via bead beating (30 s at 6.5 m s^−1^) [84].

To identify the most suitable amplification and sequencing method for this environmental sample type, we evaluated the performance of six primer pairs by amplifying either ITS1, ITS2 or the full-length ITS1-5.8S-ITS2 region of 6 patch samples (Additional file 7) [65, 85–94]. Based on the results obtained, the primer pair ITS3mix1-5/ITS4ngsUni targeting the ITS2 region was selected for investigating the fungal communities in this study. For generating ITS amplicon libraries, a two-step PCR protocol for highly multiplexed amplicon sequencing was followed in the 120 patch samples [95]. The PCR protocol and programs used are detailed in Additional file 8. Library preparation and MiSeq Illumina sequencing was performed by the Joint Microbiome Facility (JMF, University of Vienna, Austria). For sequencing, we selected a 2 × 300 bp cycles paired-end mode using the MiSeq v3 Reagent kit (Illumina).

### Sequence processing and analysis

Amplicon sequence data were processed as described in Pjevac et al. (2021) [95]. Briefly, ASVs were inferred using the DADA2 R package version 1.2.0 [96] with R v4.1.1 [97] by applying trimming at 220/230 nucleotides with allowed expected errors of 2/4. Singletons were removed from the count table. ASVs were taxonomically classified using a modified version of the UNITE v8.2 database covering eukaryotes [83, 89]. Detailed information about the sequences modified or added to the UNITE database can be found at Additional file 9 [25, 27, 30, 83, 98–103].

Downstream analyses were performed in R v4.1.2 [97] and RStudio 2021.09.1 [104]. To analyze the fungal diversity and community composition in individual ant colonies, patch samples of the 17 established colonies that were sequenced separately by tree sections were merged by adding up read counts using ampvis2 v2.7.11 R package [90]. We calculated alpha and beta-diversity analysis of fungal communities by using the R packages ampvis2 v2.7.11 [90], vegan v2.6–4 [92], and GUniFrac v1.4 [105]. For both diversity metrics, we first rarefied the read counts using the minimum read count per sample that was higher than 2000 reads. Alpha diversity metrics were analyzed calculating the Shannon index and the difference between groups was tested for statistical significance by Kruskal–Wallis test and post hoc pair-wise Wilcoxon analysis using a *p* value of 0.05. The beta diversity was visualized by PCoA using Bray–Curtis distances and statistically compared using PERMANOVA [106] and MiRKAT [107] tests with a *p* value of 0.05. Since the sample size design was notably unbalanced in most beta diversity comparisons, additional PERMDISP test [108] was performed to evaluate the heterogeneity of dispersions [109].

To inspect the fungal community composition at high taxonomic resolution (genus level), we identified the 30 most abundant ASVs and the frequent ASVs from patch samples of established colonies. Discriminative ASVs between ant species were obtained with the DESEq2 v1.34.0 R package [110] (adjusted *p* < 0.05). Furthermore, we defined frequent ASVs per each ant species when (i) they were present in at least half of the colonies of that ant species and (ii) resulted in a mean relative read abundance higher than 0.05% for such ant species. For improving legibility and accessibility, representative ASVs (abundant and frequent ASVs, and Chaetothyriales ASVs) were renamed using number digits, listed and detailed in Additional file 10. To investigate the abundance dynamics of frequent genera among different patch types (ant colony developmental stages and tree sections), we used the unmerged patch samples from established colonies and analyzed their relative abundance from all *Azteca* sp. colonies jointly. Statistical comparisons of relative abundance in each ant colony stage and tree section were calculated by Kruskal–Wallis and post hoc Wilcoxon test (*p* < 0.05).

In order to enable a comparison with the previous studies [25, 27, 47], the ASV sequences of Chaetothyriales were aligned to a representative ITS matrix of GenBank sequences of Trichomeriaceae and Cyphellophoraceae from domatia including sequences obtained from *Cecropia* by Nepel et al. (2016) and Mayer et al. (2018) [25, 47]. Details about the methodology followed for constructing such phylogenetic tree can be found in Additional file 5 [25, 47, 56–59].

### Supplementary Information

**Additional file 1.** Overview of the number of patch samples collected per each ant colony developmental stage and each ant-plant species.

**Additional file 2.** Statistical tests performed in the alpha diversity analyses (Shannon index) of fungal patch communities among different ant colony developmental stages and ant-plant species.

**Additional file 3.** Overview of reads and ASVs count per individual initial patch and per individual established colony.

**Additional file 4.** Statistical tests performed in the beta diversity analyses (Bray-Curtis distances) of fungal patch communities among different ant colony developmental stages and ant-plant species.

**Additional file 5.** Phylogenetic tree representing a monophyletic clade of domatia-inhabiting Chaetothyriales ITS sequences.

**Additional file 6.** Description of the criteria followed for dividing into sections certain *Cecropia* trees inhabited by established *Azteca* colonies.

**Additional file 7.** Performance evaluation of various ITS barcode regions, primer pairs and sequencing technologies for analyzing fungal communities in ant-made patches using culture-independent metabarcoding methods.

**Additional file 8.** PCR protocol and program used in this investigation.

**Additional file 9.** Metadata of the sequences changed or added to the modified version of the UNITE v8.2 database covering eukaryotes.

**Additional file 10.** Metadata of the most abundant and the frequent ASV sequences.

## Data Availability

All data generated or analyzed during this study are included in this published article, its supplementary information files and publicly available repositories. The sequence data (raw sequence reads and metadata) are accessible on NCBI under the BioProject accession number PRJNA777006 [111]. The ITS amplicon sequencing data supporting the conclusions of this article and the R code used for downstream analysis in this investigation are available in a collection (https://doi.org/10.6084/m9.figshare.c.7072553.v1) in the publicly available figshare repository [83]. Likewise, the modified UNITE v8.2 database used for the taxonomic assignment of ITS sequences in this study can be found in the same collection in the figshare repository [83].

## Abbreviations

ITS: Internal transcribed spacer
ASVs: Amplicon sequence variants
IP: Initial patch
YP: Young patch
EP: Established patches
EP I: Established patches from upper internodes
EP II: Established patches from intermediate internodes
EP III: Established patches from lower internodes
