# Supplementary material for: Dynamics and drivers of fungal communities in a multipartite ant-plant association
Source: BMC Biol. 2024 May 14;22:112. doi: 10.1186/s12915-024-01897-y (PMC11093746; doi:10.1186/s12915-024-01897-y)
Supplement: Supplementary file 1 — Additional file 1. Overview of the number of patch samples collected per each ant colony developmental stage and each ant-plant species. [file 12915_2024_1897_MOESM1_ESM.pdf]

Supplementary Information for:

Dynamics and drivers of fungal communities in a multipartite ant-plant association

Veronica Barrajon-Santos, Maximilian Nepel, Bela Hausmann, Hermann Voglmayr, Dagmar Woebken, Veronika E. Mayer

Additional File 1: Overview of the number of patch samples collected per each ant colony developmental stage and each ant-plant species.

Additional File 1: Table S1. Overview of number of colonies (n = 93) collected per *Azteca* ant species and per colony development stage.

	A. alfari	***A. constructor***	***A. xanthochroa***	**Total**
Initial patch (IP)	27	4	9	40
Young patch (YP)	15	2	0	17
Established patches (EP)	12	24	0	36
Total	54	30	9	**93**

Additional File 1: Table S2. Overview of number of established colonies (n = 36) collected per *Azteca* ant species and per *Cecropia* plant species.

	A. alfari	***A. constructor***	**Total**
C. peltata	8	8	16
C. obtusifolia	3	14	17
C. insignis	1	0	1
C. sp	0	2	2
Total	12	24	**36**
