# Supplementary material for: Dynamics and drivers of fungal communities in a multipartite ant-plant association
Source: BMC Biol. 2024 May 14;22:112. doi: 10.1186/s12915-024-01897-y (PMC11093746; doi:10.1186/s12915-024-01897-y)
Supplement: Supplementary file 2 — Additional file 2. Statistical tests performed in the alpha diversity analyses (Shannon index) of fungal patch communities among different ant colony developmental stages and ant-plant species. [file 12915_2024_1897_MOESM2_ESM.pdf]

Supplementary Information for:

Dynamics and drivers of fungal communities in a multipartite ant-plant association

Veronica Barrajon-Santos, Maximilian Nepel, Bela Hausmann, Hermann Voglmayr, Dagmar Woebken, Veronika E. Mayer

Additional File 2: Statistical tests performed in the alpha diversity analyses (Shannon index) of fungal patch communities among different ant colony developmental stages and ant-plant species.

Additional File 2: Table S1. Statistical analysis of the fungal alpha diversity Shannon index comparison among patch samples of *A. alfari* colonies at different developmental stages.

**Summary stats**	**n**	**mean**	**sd**
Initial patch (IP) samples	27	1.17	0.53
Young patch (YP) samples	15	1.65	0.47
Established patches (EP) samples	12	2.11	0.67
**Kruskal-Wallis test**	**parameter**	**statistic**	***p***
	2	16.42	0.00027
**pairwise Wilcoxon test**			***p***
IP-YP			0.0073
IP-EP			0.0008
YP-EP			0.0469

Additional File 2: Table S2. Statistical analysis of the fungal alpha diversity Shannon index comparison among patch samples of *A. constructor* colonies at different developmental stages.

**Summary stats**	**n**	**mean**	**sd**
Initial patch (IP) samples	4	1.43	0.72
Young patch (YP) samples	2	0.73	0.53
Established patches (EP) samples	24	2.35	0.54
**Kruskal-Wallis test**	**parameter**	**statistic**	***p***
	2	10.15	0.0062
**pairwise Wilcoxon test**			***p***
IP-YP			0.2667
IP-EP			0.0227
YP-EP			0.0185

Additional File 2: Table S3. Statistical analysis of the fungal alpha diversity Shannon index comparison among established patch samples of *A. alfari* colonies inhabiting *C. peltata* and *C. obtusifolia* trees.

Summary stats	n	mean	sd
C. peltata	8	1.967	0.787
C. obtusifolia	3	2.431	0.317
**Kruskal-Wallis test**	**parameter**	**statistic**	***p***
	1	1.042	0.307

Additional File 2: Table S4. Statistical analysis of the fungal alpha diversity Shannon index comparison among established patch samples of *A. constructor* colonies inhabiting *C. peltata* and *C. obtusifolia* trees.

Summary stats	n	mean	sd
C. peltata	8	2.095	0.585
C. obtusifolia	14	2.518	0.445
**Kruskal-Wallis test**	**parameter**	**statistic**	***p***
	1	3.149	0.076
