# Supplementary material for: Dynamics and drivers of fungal communities in a multipartite ant-plant association
Source: BMC Biol. 2024 May 14;22:112. doi: 10.1186/s12915-024-01897-y (PMC11093746; doi:10.1186/s12915-024-01897-y)
Supplement: Supplementary file 3 — Additional file 3. Overview of reads and ASVs count per individual initial patch and per individual established colony. [file 12915_2024_1897_MOESM3_ESM.pdf]

Supplementary Information for:

Dynamics and drivers of fungal communities in a multipartite ant-plant association

Veronica Barrajon-Santos, Maximilian Nepel, Bela Hausmann, Hermann Voglmayr, Dagmar Woebken, Veronika E. Mayer

Additional File 3: Overview of reads and ASVs count per individual initial patch and per individual established colony.

Additional File 3: Table S1. Mean fungal reads count and mean fungal ASV count per individual initial patch (IP) and per individual established colony (EP) including the standard deviation (SD) and standard error (SE). Moreover, the mean number of ASVs that accounted for 90% of reads per colony is shown.

	**Reads #**		**ASVs #**		**ASVs # representing 90% Reads**	
	IP	EP	IP	EP	IP	EP
**Mean**	20546	24662	31	189	4	15
**SD**	12423	15178	14	77	2	11
**SE**	1964	2566	2	13	0	2
