# Supplementary material for: Dynamics and drivers of fungal communities in a multipartite ant-plant association
Source: BMC Biol. 2024 May 14;22:112. doi: 10.1186/s12915-024-01897-y (PMC11093746; doi:10.1186/s12915-024-01897-y)
Supplement: Supplementary file 4 — Additional file 4. Statistical tests performed in the beta diversity analyses (Bray-Curtis distances) of fungal patch communities among different ant colony developmental stages and ant-plant species. [file 12915_2024_1897_MOESM4_ESM.pdf]

Supplementary Information for:

Dynamics and drivers of fungal communities in a multipartite ant-plant association

Veronica Barrajon-Santos, Maximilian Nepel, Bela Hausmann, Hermann Voglmayr, Dagmar Woebken, Veronika E. Mayer

Additional File 4: Statistical tests performed in beta diversity analyses (Bray-Curtis distances) of fungal patch communities among different ant colony developmental stages and ant-plant species.

Additional File 4: Table S1. Statistical analysis of the fungal beta diversity (Bray-Curtis) comparison among initial patches (IP) of *A. alfari*, *A. constructor* and *A. xanthochroa* colonies.

**PERMANOVA test**	**Df**	**F. Model**	**R2**	**Pr (>F)**
Groups	2	1.1063	0.058	0.197
Residuals	36		0.942	
**PERMANOVA pairwise test**	**F. Model**	**R2**	***p***	***p. adjust***
A. alfari – A. constructor	1.413	0.046	0.203	0.339
A. alfari – A. xanthochroa	0.372	0.011	0.863	0.869
A. constructor – A. xanthochroa	1.798	0.152	0.108	0.249
**PERMDISP test**	**Df**	**F. Model**	**N. Perm**	**Pr (>F)**
Groups	2	1.853	999	0.190
Residuals	36			
**PERMDISP pairwise test**			**observed *p***	**permuted *p***
A. alfari – A. constructor			0.083	0.072
A. alfari – A. xanthochroa			0.295	0.306
A. constructor – A. xanthochroa			0.447	0.434
**MiRKAT test**				***p***
A. alfari – A. constructor				0.059

Additional File 4: Table S2. Statistical analysis of the fungal beta diversity (Bray-Curtis) comparison among established patches (EP) of *A. alfari* and *A. constructor*.

**PERMANOVA test**	**Df**	**F. Model**	**R2**	**Pr (>F)**
Groups	1	3.491	0.093	0.001
Residuals	34		0.907	
**PERMDISP test**	**Df**	**F. Model**	**N. Perm**	**Pr (>F)**
Groups	1	0.608	999	0.439
Residuals	34			
**MiRKAT test**				***p***
A. alfari – A. constructor				0.001

Additional File 4: Table S3. Statistical analysis of the fungal beta diversity (Bray-Curtis) comparison among established patches (EP) of *A. alfari* inhabiting *C. peltata* and *C. obtusifolia* trees.

**PERMANOVA test**	**Df**	**F. Model**	**R2**	**Pr (>F)**
Groups	1	1.035	0.103	0.342
Residuals	9		0.897	
**PERMDISP test**	**Df**	**F. Model**	**N. Perm**	**Pr (>F)**
Groups	1	0.028	999	0.875
Residuals	9			
**MiRKAT test**				***p***
C. peltata - C. obtusifolia				0.326

Additional File 4: Table S4. Statistical analysis of the fungal beta diversity (Bray-Curtis) comparison among established patches (EP) of *A. constructor* inhabiting *C. peltata* and *C. obtusifolia* trees.

**PERMANOVA test**	**Df**	**F. Model**	**R2**	**Pr (>F)**
Groups	1	1.366	0.064	0.059
Residuals	20		0.936	
**PERMDISP test**	**Df**	**F. Model**	**N. Perm**	**Pr (>F)**
Groups	1	3.900	999	0.059
Residuals	20			
**MiRKAT test**				***p***
C. peltata - C. obtusifolia				0.05
