# Supplementary material for: Dynamics and drivers of fungal communities in a multipartite ant-plant association
Source: BMC Biol. 2024 May 14;22:112. doi: 10.1186/s12915-024-01897-y (PMC11093746; doi:10.1186/s12915-024-01897-y)
Supplement: Supplementary file 5 — Additional file 5. Phylogenetic tree representing a monophyletic clade of domatia-inhabiting Chaetothyriales ITS sequences. [file 12915_2024_1897_MOESM5_ESM.pdf]

Supplementary Information for:

Dynamics and drivers of fungal communities in a multipartite ant-plant association

Veronica Barrajon-Santos, Maximilian Nepel, Bela Hausmann, Hermann Voglmayr, Dagmar Woebken, Veronika E. Mayer

Additional File 5: Phylogenetic tree representing a monophyletic clade of domatia-inhabiting Chaetothyriales ITS sequences.

Additional File 5: Methods.

In order to enable a comparison with the previous analyses of Nepel et al. (2016) [25] and Mayer et al. (2018) [47], the ASV sequences of Chaetothyriales were aligned to a representative ITS matrix of GenBank sequences of Trichomeriaceae and Cyphellophoraceae from domatia including sequences obtained from *Cecropia* by Nepel et al. (2016) [25] and Mayer et al. (2018) [47]. For phylogenetic analyses, sequences were aligned with the server version of MAFFT v. 7.490 [57], checked and refined using BioEdit v7.2.6 [56]. Maximum likelihood (ML) analyses were performed with RAxML [59] as implemented in raxmlGUI v2.0.9 [58], using the ML + rapid bootstrap setting and the GTRGAMMA substitution model with 1000 bootstrap replicates.

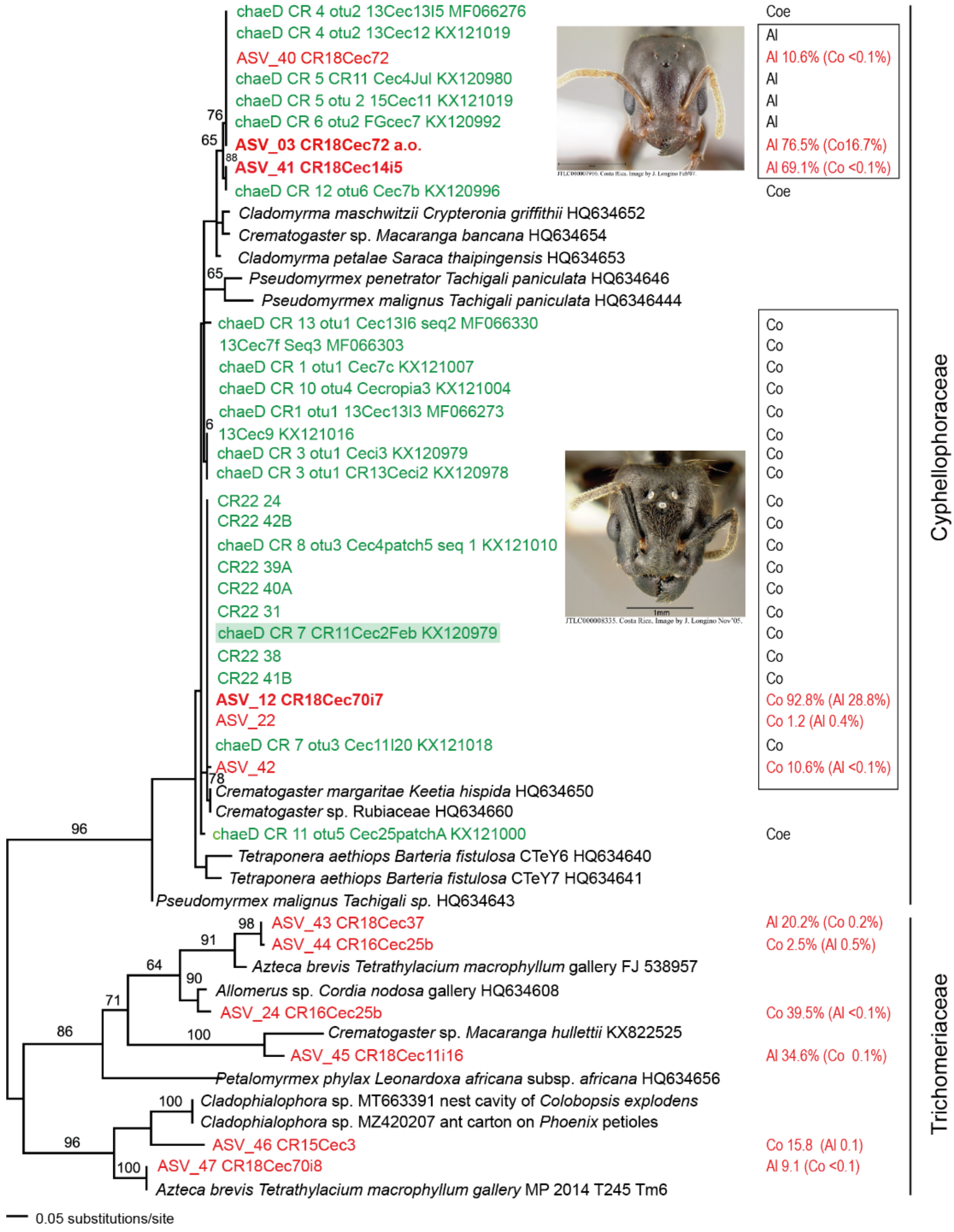

Additional File 5: Figure S1. Maximum likehood tree of Cyphellophoraceae and Trichomeraceae ITS sequences from this study (red), and previous investigations of *Azteca-Cecropia* association (green) as well as of other ant-plant associations (black) with the respective Genbank accession number. The ant species of from which the samples were taken is listed on the right side (Al: *A. alfari*, Co: *A. constructor*, Coe: *A. coeruleipennis*). Percentages give the maximum read relative abundance of that sequence. The sample from which the genome was analysed (CBS 132003) is highlighted in green. Values above branches represent 12ML bootstrap support above 60%. Photos: by Jack Longino.
