# Supplementary material for: Dynamics and drivers of fungal communities in a multipartite ant-plant association
Source: BMC Biol. 2024 May 14;22:112. doi: 10.1186/s12915-024-01897-y (PMC11093746; doi:10.1186/s12915-024-01897-y)
Supplement: Supplementary file 6 — Additional file 6. Description of the criteria followed for dividing into sections certain Cecropia trees inhabited by established Azteca colonies. [file 12915_2024_1897_MOESM6_ESM.pdf]

Supplementary Information for:

Dynamics and drivers of fungal communities in a multipartite ant-plant association

Veronica Barrajon-Santos, Maximilian Nepel, Bela Hausmann, Hermann Voglmayr, Dagmar Woebken, Veronika E. Mayer

Additional File 6: Description of the criteria followed for dividing into sections certain *Cecropia* trees inhabited by established *Azteca* colonies.

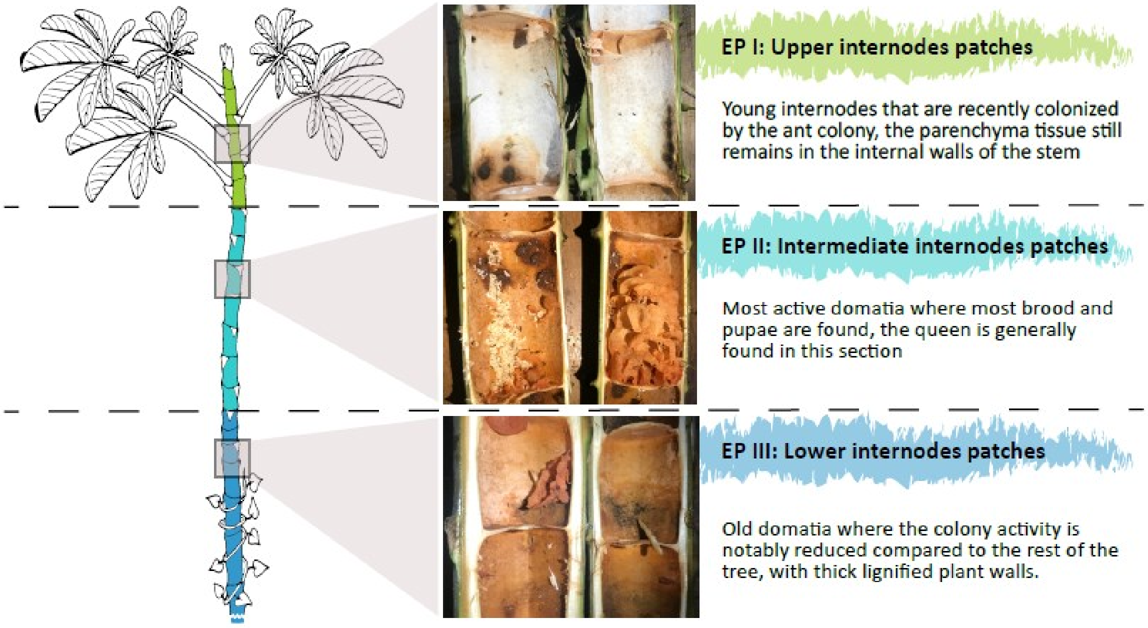

Additional File 6: Figure S1. Illustration of established *Azteca* ant colony distribution in a *Cecropia* tree. For 17 established colonies, patches were subsampled by differentiating three stem sections.
