# Supplementary material for: Dynamics and drivers of fungal communities in a multipartite ant-plant association
Source: BMC Biol. 2024 May 14;22:112. doi: 10.1186/s12915-024-01897-y (PMC11093746; doi:10.1186/s12915-024-01897-y)
Supplement: Supplementary file 7 — Additional file 7. Performance evaluation of various ITS barcode regions, primer pairs and sequencing technologies for analyzing fungal communities in ant-made patches using culture-independent metabarcoding methods. [file 12915_2024_1897_MOESM7_ESM.pdf]

Supplementary Information for:

Dynamics and drivers of fungal communities in a multipartite ant-plant association

Veronica Barrajon-Santos, Maximilian Nepel, Bela Hausmann, Hermann Voglmayr, Dagmar Woebken, Veronika E. Mayer

Additional File 7: Performance evaluation of various ITS barcode regions, primer pairs and sequencing technologies for analysing fungal communities in ant-made patches using culture-independent metabarcoding methods.

Additional File 7: Methods

In metabarcoding analysis, the selection of a suitable genetic marker, primer pair and thermocycling conditions are some of the most important steps for obtaining a complete and correct representation of fungal communities in a certain environment [65, 85]. Specially for understudied and highly diverse ecosystems like the Tropics, the risk of using a primer pair that either present mismatches with any of the most relevant fungal groups or amplifies untargeted DNA from non-fungal organisms is eminent. For obtaining high quality amplicons, a prior optimization step where different genetic markers and primer pairs are tested is crucial.

In this study, we aimed to elucidate the diversity and taxonomic composition of fungal communities inhabiting patches of an ant-plant mutualism in the Tropics of Central America. To identify the most suitable amplification and sequencing method for this environmental sample type, we evaluated: i) genetic markers: ITS1, ITS2 and full-length ITS1-5.8S-ITS2 region; ii) primer pairs: ITS1Fngs-ITS2, ITS1Fng-gITS7ngsRev, gITS7ngs-ITS4ngsUni, ITS3mix-ITS4ngsUni, ITS1catta-ITS4ngsUni and ITS9MUNngs-ITS4ngsUni; and, iii) sequencing strategies: Next Generation Sequencing (NGS) Illumina Sequencing (Illumina Inc., San Diego, USA), and Third Generation Sequencing (TGS) PacBio Sequencing (Pacific Bioscience, CA, USA). Details about the primer pairs selected can be found at Additional File 7: Table S1. The DNA extraction method used in this test was the same as described in Material and Methods of this manuscript. For each primer pair, a negative control without DNA aliquot was amplified and sequenced parallelly to the six patch samples.

Samples sequenced by PacBio were amplified by triplicates using the following PCR program: 95 °C for 15 min followed by 28 cycles of 95 °C for 30 s, 55 °C for 30 s, 72°C for 1 min; and final 72°C for 10 min [87, 88]. Replicates were cleaned up using AMPure XP magnetic beads (Beckman Coulter, Germany) in a bead to sample ratio of 0.7x and pooled. Prior to equimolarly pooling of both samples, DNA concentrations were measured using Quant-iT PicoGreen. The SMRTbell library preparation and sequencing using Sequel SMRTcell 20hrs (both, Pacific Biosciences, CA, USA) were performed by the Vienna Biocenter Core Facilities (VBCF; Vienna, Austria). PacBio Circular Consensus Sequences (CCS) were generated using ccs 4.0.0 (minPasses = 3, MinAccuracy = 0.9) from pbbioconda v2.2.2. Complement-reverse sequences were generated from reads that were sequenced inversely by using primer detection fqgrep v0.4.4 tool and fastxtoolkit v0.0.14. Subsequent reads were quality checked and trimmed by vsearch v2.14.1 [89] using the following parameters: fastq_qmax = 93, fastq_maxee = 1, fastq_maxns = 0. Minimum sequences length was set as 400 bp for ITS. From vsearch software, full-

length de-replication and de novo chimera removal were applied to reads before clustering (by cluster size) into operational taxonomic units (OTUs) at 97% sequence similarity [65].

Samples for Illumina sequencing were processed and sequenced as described in Material and Methods of this manuscript. Paired-end sequence data from Illumina sequencing was pre-processed and quality filtered as described in Material and Methods of this manuscript. After quality filtering, we merged pair-end sequences using usearch v11. Then, we followed the same OTU-based clustering pipeline from vsearch as described for PacBio sequencing data instead of following the DADA2 pipeline used for the big Illumina dataset.

Fungal OTUs representatives from all primer pairs were taxonomically classified by CONSTAX v2 using a consensus classification obtained from RDP, Blast and SINTAX classifiers against the modified version of the UNITE v8.2 database covering eukaryotes [83, 90]. Downstream analysis of sequence data was performed by using the R packages ampvis2 v2.7.11 [91], rstatix v0.8.0 [92] and vegan v2.6-4 [93]. To perform the primer pair comparisons, ampvis2 objects from each primer pair dataset were separately created and subsequently merged by using amp_merge_ampvis2 command. For Chao1 and Shannon alpha-diversity, we first rarefied the read counts using the minimum read count per sample that was higher than 2000 reads. Samples with less than 2000 reads were excluded for diversity metrics. Statistical significance among primers was tested by ANOVA and subsequent TukeyHSD post-hoc analysis using a p value of 0.05 for alpha diversity metrics and by Kruskal-Wallis and post-hoc Wilcoxon test for recovering relative abundances of eukaryote kingdoms and classes ($p < .05$).

Additional File 7: Results

By looking at the total read count per sample, we observed that the PCR and sequencing performance of primer pair gITS7ngs-ITS4ngsUni targeting ITS2 region was suboptimal, resulting in strongly varying number of reads among tested patch samples (Additional File 7: Table S2). Overall, classification ratios (85-98% among primer pairs) and confidence values (>96% for all primer pairs) at genus level did not significantly vary among primer pairs (data not shown). As this type of environmental samples harbors a wide diversity of organisms apart from fungi, we tested the performance of each primer pair in discriminating against non-fungal DNA (Additional File 7: Figure S3). Primer pair ITS9MUNngs-ITS4ngsUni used in combination with PacBio-sequencing (780 bp long reads) was highly inefficient for discriminating against nematodes DNA of these patches compared to the other primer pairs, leading to a high proportion of reads assigned to metazoan taxa. Similarly, primer pair ITS1catta-ITS4ngsUni (630 bp long reads) was inefficient for discriminating against Protista DNA. In consequence, the proportion of fungal reads from these primer pairs resulted in the lowest of all primer pairs tested, with 51% mean relative abundance and 82% mean relative abundance of kingdom Fungi for ITS9MUNngs-ITS4ngsUni and ITS1catta-ITS4ngsUni, respectively.

For further evaluation, we compared the relative read abundances of the abundant fungal classes (>1% mean relative read abundance) in the tested patch samples among primer pairs (Additional File 7: Figure S2). Overall, relative abundances did not differ significantly among the six primer pairs tested except for: i) unclassified fungal sequences that were significantly more abundant in primer pair ITS1Fngs-ITS2 than the ITS2 primer pairs; and, ii) Mortierellomycetes sequences that were less abundant in primer pair ITS1catta-ITS4ngsUni (PacBio sequencing) than the primer pairs used for Illumina sequencing. Regarding alpha diversity metrics, no significant differences were detected either between primers (Additional File 7: Figure S1).

The latest recommendations for fungal microbiome studies highlighted the biases caused by the ITS1 regions and specifically, by the ITS1F-ITS2 primer pair. In fact, Tedersoo et al. (2022) recently underlined that primer ITS1F is particularly problematic because of several critical mismatches in certain groups of moulds and putative animal pathogens [85]. As it is highly important for our research to detect the presence of entomopathogenic fungal groups, we decided to discard this primer pair. In conclusion, based on the results obtained in this primer pair comparison and the current recommendations for fungal microbiome studies [65, 85, 87, 94] the primer pair ITS3mix1-5/ITS4ngsUni targeting the ITS2 region was selected for investigating the fungal community in this study.

Additional File 7: Table S1. Primers and sequencing methods used for primer pair comparison.

Primer pair	Gene target	Seq. method	Seq. length (bp)	Forward primer	Reverse primer	References
ITS1Fngs - ITS2	ITS1	Illumina MiSeq	320	GGTCATTTAGAGGAAGTAA	GCTGCGTTCTTCATCGATGC	Tedersoo et al., 2015; White et al., 1999 [93, 94]
ITS1Fngs - gITS7ngsRev	ITS1	Illumina MiSeq	380	GGTCATTTAGAGGAAGTAA	CAAARAYTYGATGAYTCAC	Tedersoo & Lindahl, 2016; this study [86]
gITS7ngs - ITS4ngsUni	ITS2	Illumina MiSeq	350	GTGARTCATCRARTYTTTG	CCTSCSCTTANTDATATGC	Tedersoo & Lindahl, 2016; Tedersoo et al., 2015 [86, 93]
ITS3mix - ITS4ngsUni	ITS2	Illumina MiSeq	401	CATCGATGAAGAACGTRG	CCTSCSCTTANTDATATGC	Tedersoo et al., 2015 [93]
ITS1catta - ITS4ngsUni	FULL ITS	PacBio CCS	630	ACCWGCGGARGGATCATTA	CCTSCSCTTANTDATATGC	Tedersoo et al., 2019; Tedersoo et al., 2015 [87, 93]
ITS9MUNngs - ITS4ngsUni	FULL ITS	PacBio CSS	780	TACACACCGCCCGTCG	CCTSCSCTTANTDATATGC	Tedersoo & Lindahl, 2016; Tedersoo et al., 2015 [86, 93]

Additional File 7: Table S2. Read counts obtained in each sequenced sample per primer pair.

Primer pair	Sample ID	Sample name	Read count
ITS1Fngs_ITS2	sample_67	CR16_24_Ep_III_t0_E6	358513
ITS1Fngs_ITS2	sample_69	CR16Cec19_Ep_III_t0_E8	54717
ITS1Fngs_ITS2	sample_73	CR16Cec8_Ep_I_t0	40600
ITS1Fngs_ITS2	sample_75	CR16Cec26_27_Ep_t0	41667
ITS1Fngs_ITS2	sample_77	Cr16Cec28_29_Ep_pool_t0	4305
ITS1Fngs_ITS2	sample_79	CR18Cec14i5_Fp_t0	18155
ITS1Fngs_gITS7ngsRev	sample_51	CR16_24_Ep_III_t0_E6	138428
ITS1Fngs_gITS7ngsRev	sample_53	CR16Cec19_Ep_III_t0_E8	48227
ITS1Fngs_gITS7ngsRev	sample_57	CR16Cec8_Ep_I_t0	26809
ITS1Fngs_gITS7ngsRev	sample_59	CR16Cec26_27_Ep_t0	25596
ITS1Fngs_gITS7ngsRev	sample_61	Cr16Cec28_29_Ep_pool_t0	**379**
ITS1Fngs_gITS7ngsRev	sample_63	CR18Cec14i5_Fp_t0	8605
gITS7ngs_ITS4ngsUni	sample_19	CR16_24_Ep_III_t0_E6	198227
gITS7ngs_ITS4ngsUni	sample_21	CR16Cec19_Ep_III_t0_E8	18570
gITS7ngs_ITS4ngsUni	sample_25	CR16Cec8_Ep_I_t0	**551**
gITS7ngs_ITS4ngsUni	sample_27	CR16Cec26_27_Ep_t0	20230
gITS7ngs_ITS4ngsUni	sample_29	Cr16Cec28_29_Ep_pool_t0	**815**
gITS7ngs_ITS4ngsUni	sample_31	CR18Cec14i5_Fp_t0	**593**
ITS3mix_ITS4ngsUni	sample_11	CR16Cec26_27_Ep_pool_t0	47339
ITS3mix_ITS4ngsUni	sample_13	Cr16Cec28_29_Ep_pool_t0	21912
ITS3mix_ITS4ngsUni	sample_15	CR18Cec14i5_Fp_t0	8548
ITS3mix_ITS4ngsUni	sample_3	CR16_24_Ep_III_t0_E6	11647
ITS3mix_ITS4ngsUni	sample_5	CR16Cec19_Ep_III_t0_E8	6560
ITS3mix_ITS4ngsUni	sample_9	CR16Cec8_Ep_I_t0	4911
ITS1catta_ITS4ngsUni	sample_021	CR16_24_Ep_III_t0_E6	5829
ITS1catta_ITS4ngsUni	sample_029	CR16Cec19_Ep_III_t0_E8	4918
ITS1catta_ITS4ngsUni	sample_032	CR16Cec8_Ep_I_t0	2359
ITS1catta_ITS4ngsUni	sample_034	CR16Cec26_27_Ep_t0	2101
ITS1catta_ITS4ngsUni	sample_038	CR18Cec14i5_Fp_t0	1268
ITS1catta_ITS4ngsUni	sample_190	CR16Cec28_29_Ep_t0	2008
ITS9MUNngs_ITS4ngsUni	sample_025	CR16Cec8_Ep_I_t0	6413
ITS9MUNngs_ITS4ngsUni	sample_251	CR18Cec14i5_Fp_t0	3883
ITS9MUNngs_ITS4ngsUni	sample_330	CR16Cec19_Ep_III_t0_E8	8789
ITS9MUNngs_ITS4ngsUni	sample_338	CR16Cec26_27_Ep_t0	5773
ITS9MUNngs_ITS4ngsUni	sample_339	CR16_24_Ep_III_t0_E6	5483
ITS9MUNngs_ITS4ngsUni	sample_400	CR16Cec28_29_Ep_t0	4898

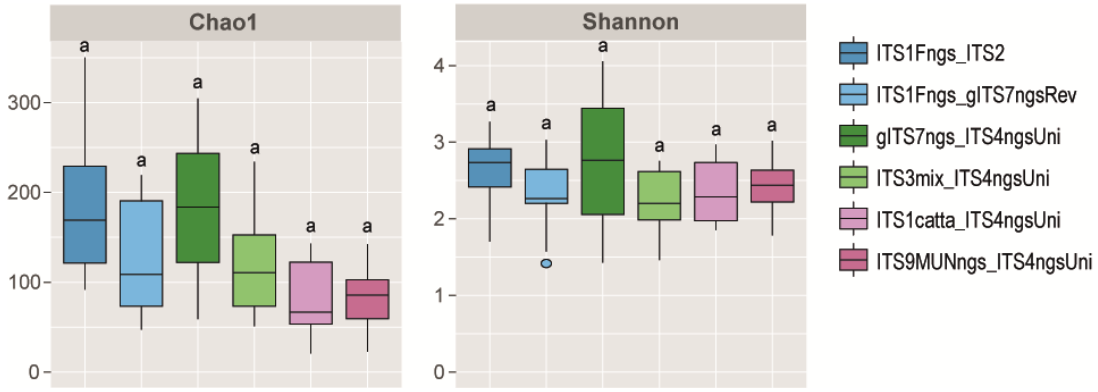

Additional File 7: Figure S1. Alpha diversity of fungal communities using different primer pairs. Left: richness (Chao1); right: Shannon Index. Statistical comparisons are calculated by ANOVA and post-hoc Tukey test ($p < .05$).

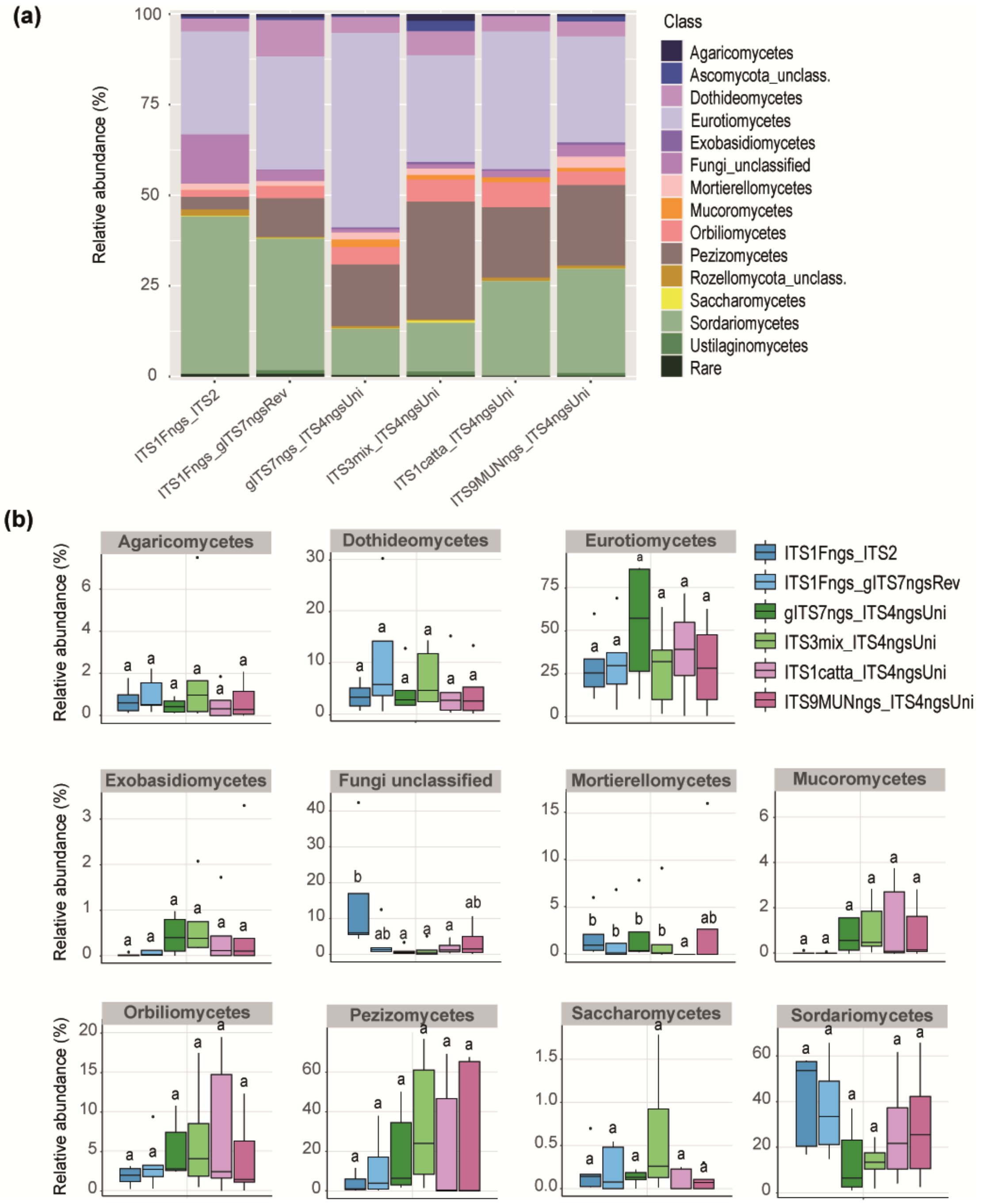

Additional File 7: Figure S2. Relative read abundances of fungal classes detected in six patch samples when using different primer pairs. (a) Taxonomic distribution showing mean relative read abundances of fungal classes with tested primer pairs. (b) Statistical comparisons (Kruskal-Wallis and post-hoc Wilcoxon test) of primers pairs for recovering fungal classes ($p < .05$).

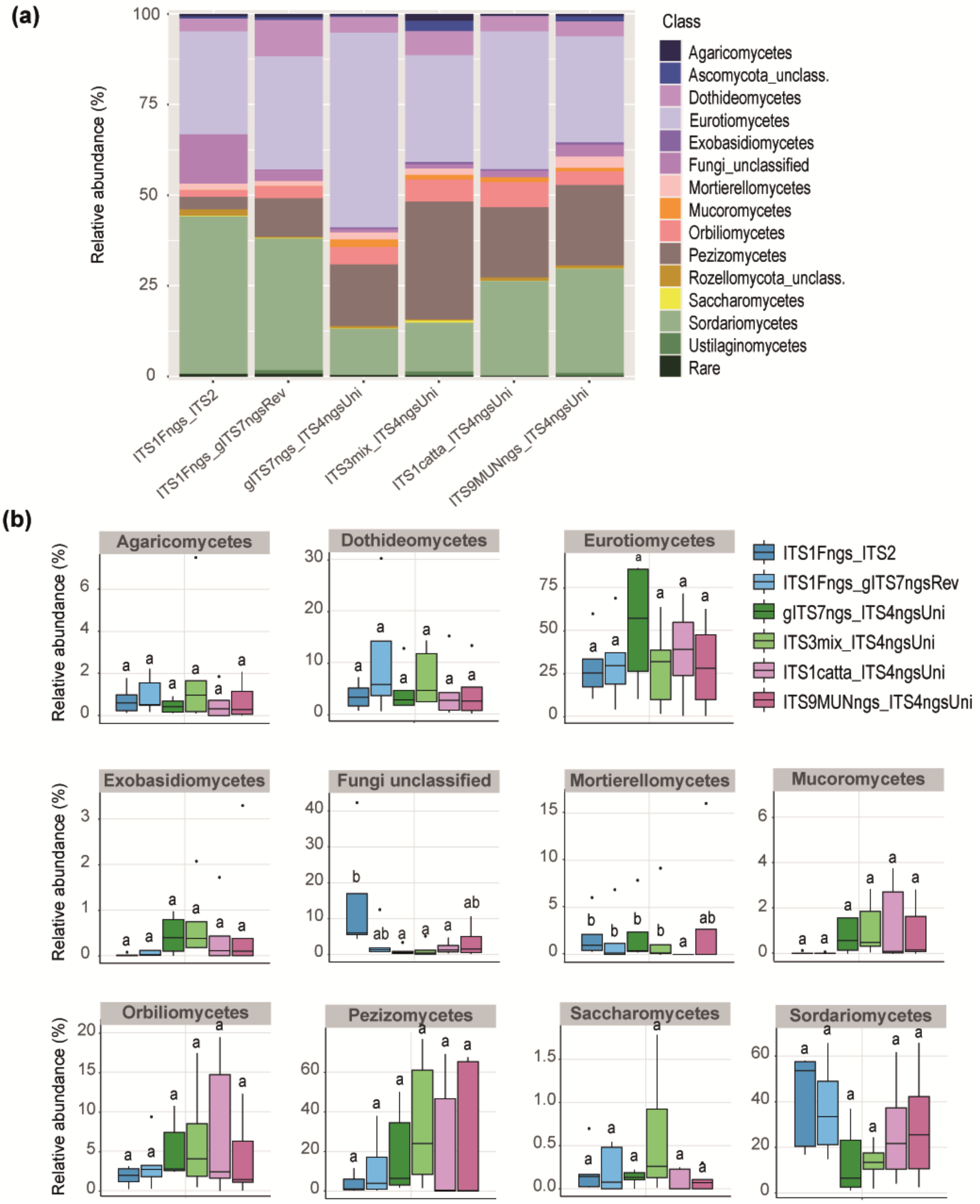

Additional File 7: Figure S3. Relative read abundances of eukaryote kingdoms detected in six patch samples when using different primer pairs. (a) Taxonomic distribution showing mean relative read abundances of kingdoms with tested primer pairs. (b) Statistical comparisons (Kruskal-Wallis and post-hoc Wilcoxon test) of used primers pairs for amplifying diverse eukaryote kingdoms ($p < .05$).
