# Supplementary material for: Dynamics and drivers of fungal communities in a multipartite ant-plant association
Source: BMC Biol. 2024 May 14;22:112. doi: 10.1186/s12915-024-01897-y (PMC11093746; doi:10.1186/s12915-024-01897-y)
Supplement: Supplementary file 8 — Additional file 8. PCR protocol and program used in this investigation. [file 12915_2024_1897_MOESM8_ESM.pdf]

Supplementary Information for:

Dynamics and drivers of fungal communities in a multipartite ant-plant association

Veronica Barrajon-Santos, Maximilian Nepel, Bela Hausmann, Hermann Voglmayr, Dagmar Woebken, Veronika E. Mayer

Additional File 8: PCR protocol and program used in this investigation

Additional File 8: Methods

In the first step PCR, 3 µL of DNA (2 ng/µL) were added to a 25 µL PCR reaction in triplicates per patch sample. After 30 cycles of amplification, the triplicates were pooled and the PCR product was normalized with a SEQPREP Normalization Plate Kit 96 (Thermo Fisher Scientific, A1051001). In the second step PCR (barcoding), 10 µL of the PCR product from the first step PCR were added to a 50 µL PCR reaction. After 8 cycles of amplification, the normalization of the PCR product was performed again. Subsequently, all samples were pooled and purified by the innuPREP PCR pure Kit (Analytic Jena, 845-KS-5010250).

Additional File 8: Table S1. PCR program used for the amplification of DNA from patch samples.

PCR program	Cycles	T (°C)	Time	PCR program	Cycles	T (°C)	Time
1st step PCR	1x	94	4 min	2nd step PCR	1x	94	4 min
	30x	94	30 sec		7x	94	30 sec
		55	45 sec			52	30 sec
		72	1 min			72	1 min
	1x	72	10 min		1x	72	7 min
		4	∞			4	∞
